# Correlation between Immunohistochemical Markers in Hepatocellular Carcinoma Cells and *In Vitro* High-Throughput Drug Sensitivity Screening

**DOI:** 10.1155/2022/5969716

**Published:** 2022-01-25

**Authors:** Guo-Ying Feng, Yu Cheng, Kai Chen, Zheng-Rong Shi

**Affiliations:** ^1^Department of Hepatobiliary Surgery, The First Affiliated Hospital of Chongqing Medical University, Chongqing, China; ^2^Nursing Department, University-Town Hospital of Chongqing Medical University, Chongqing, China

## Abstract

**Aim:**

This study analyzed the correlation between immunohistochemical markers in hepatocellular carcinoma cells and the results of *in vitro* high-throughput drug sensitivity screening, to provide a reference for individualized drug treatment in patients with liver cancer.

**Methods:**

Seventy-four patients with hepatocellular carcinoma were included in this study from December 2019 to June 2021, and their liver cancer cells were used for *in vitro* high-throughput drug sensitivity screening. According to the screening results, the patients were divided into relatively sensitive and insensitive groups, and the correlations between sensitivity and immunohistochemistry results were analyzed statistically.

**Results:**

Alpha-fetoprotein (AFP)-positive liver cancer cells were significantly more sensitive to gemcitabine than AFP-negative cells (*χ*^2^ = 6.102, *P*=0.014). AFP was also positively correlated with sensitivity of liver cancer cells to three combined regimens containing oxaliplatin (L-OHP) and epirubicin (EPI) : L-OHP + EPI + irinotecan + 5-fluorouracil (5-FU), L-OHP + irinotecan + EPI, and L-OHP + EPI (*χ*^2^ = 8.168, *P*=0.004, *χ*^2^ = 5.705, *P*=0.017, and *χ*^2^ = 8.275, *P*=0.004, respectively).

**Conclusion:**

Gemcitabine and L-OHP + EPI + irinotecan + 5-FU, L-OHP + EPI, and L-OHP + irinotecan + EPI were more effective against AFP-positive compared with AFP-negative liver cancer cells according to *in vitro* high-throughput drug sensitivity screening. These results may guide the selection of personalized drug treatments for patients with advanced liver cancer in the future but still need further clinical studies to confirm.

## 1. Introduction

According to [1], HCC is the sixth most common cancer globally and the fourth leading cause of cancer-related death. Liver resection, liver transplantation, and liver tumor ablation are potentially curative treatments for liver cancer patients with BCLC-0 and A [2]. However, its latent occurrence and lack of obvious clinical symptoms, together with the patient's lack of awareness of the need for physical examination and follow-up, mean that more than half of all HCC patients are already in the intermediate, advanced, or terminal stages of BCLC at diagnosis. Multiple or giant tumors and accompanying metastases make surgical treatment impossible [3], and the remaining options in these patients include palliative, down-staging, or systemic treatments, such as transcatheter arterial chemoembolization (TACE), hepatic artery infusion chemotherapy (HAIC), radiofrequency and microwave ablation, and targeted therapy and immunotherapy [2, 4]. The single or combined use of these treatments (e.g., TACE combined with sorafenib [5] or apatinib [6], HAIC combined with sorafenib [7]) has achieved some clinical results; however, the benefits in patients with advanced liver cancer are still not optimistic.

There are several reasons for the poor treatment outcomes in patients with advanced liver cancer, including the natural drug resistance of liver cancer cells and the high expression of various proteins, such as P-glycoprotein and SSX2IP, which promote drug resistance [8, 9]. As a result, even the high liver concentrations of drugs achieved by HAIC fail to produce satisfactory results. The heterogeneity of liver cancer is also an important factor contributing to the unsatisfactory outcomes. Chronic liver inflammation and liver fibrosis due to various causes result in liver cancer with extremely abundant environmental susceptibility. This susceptibility, together with genetic diversity due to genome instability, leads to a high degree of heterogeneity and the unique molecular characteristics of liver cancer. This heterogeneity may be reflected in the histological types (e.g., thin trabecular type, thick trabecular type, pseudoglandular type, and compact type), as well as in the cellular phenotype (e.g., clear-cell type, fat-rich type, spindle-cell type, and undifferentiated type). It may also be reflected in terms of the tumor differentiation status and growth pattern (e.g., invasion of ambient normal liver tissue, invasion of the capsule, generation of satellite nodules, intrahepatic metastasis, and formation of tumor thrombi) [10]. Differences in the microenvironment during tumorigenesis and development also affect the plasticity and heterogeneity of cancer cells [11]. Overall, the above factors may contribute to the unsatisfactory effects of systemic therapy for liver cancer. More precise and individualized treatments are therefore needed to improve the outcomes in patients with liver cancer. High-throughput drug sensitivity screening (HDS) is a precision medication technology system for cancer patients. This approach overcomes the core technical problem of difficulties in cell isolation and culture in traditional drug susceptibility tests and uses an improved conditionally reprogrammed cell technology to achieve the rapid expansion of tumor cells. The technique uses a specially designed culture medium to simulate the tumor microenvironment and maintain the genetic and drug sensitivity characteristics of primary tumor cells *in vitro* and tumor cells *in vivo*. This method allows the simultaneous screening of 100–1,000 drugs, with a wide screening range (covering all drugs approved by the FDA), and can thus provide comprehensive drug testing and precise treatment for patients with early/middle/late-stage cancer in a short period of time. A previous study [12] showed that HDS was a safe and reliable method for helping to select postoperative adjuvant chemotherapy drugs for liver cancer patients, compared with empirical chemotherapy; however, this approach is still not widely used in clinical practice. In this study, we examined the HDS results for several cytotoxic and targeted drugs and chemotherapy regimens in relation to the immunohistochemical characteristics of liver cancer cells, to identify possible correlations as a reference for clinical treatment.

## 2. Materials and Methods

### 2.1. Study Patients

The study included 74 patients with HCC who underwent surgery at the Liver Center of the First Affiliated Hospital of Chongqing Medical University from December 2019 to June 2021. The inclusion criteria were as follows: (1) ages 18–75 years, with preoperative clinical data, test reports, and imaging reports, initially diagnosed with primary liver cancer; (2) recurrence of HCC after surgery; (3) surgical indications for complete liver tumor removal; and (4) postoperative pathological diagnosis of HCC. The exclusion criteria were (1) ages <18 years or >75 years; (2) metastatic liver cancer or liver cancer combined with other malignant tumors; (3) no indications for surgery; (4) unable to obtain enough samples for primary tumor cell culture; (5) liver cancer tissues contaminated due to various factors (e.g., sampling, transportation, culture), and primary liver cancer cells cannot be obtained; (6) unable to obtain HDS results for reasons such as culture failure; and (7) patients who refused to join the study.

### 2.2. Ethics and Informed Consent

The study complied with the ethical guidelines of the Declaration of Helsinki revised in 1975 and was approved by the Ethics Committee of the First Affiliated Hospital of Chongqing Medical University (No. 2019–021). The study was registered with the China Clinical Trial Center (https://www.chictr.org.cn; registration no. ChiCTR1900022193). A dedicated person provided the patient or their designated agent with complete and comprehensive written information about the purpose, procedures, and possible risks of this study before enrollment and informed them that they had the right to withdraw from the study at any time. Patients could be included in the study after providing written informed consent.

### 2.3. *In Vitro* HDS

#### 2.3.1. Treatment of Tumor Specimens

Liver tumor specimens obtained during surgery were placed in transport solution and transported under refrigerated conditions at 4°C. The sample delivery time was <48 h. The samples were digested to obtain primary tumor cells. To maintain consistency among the clinical samples, only one *in vitro* amplification was performed. Cells were counted after adding 10 *μ*L of the cell suspension to RPMI 1640 medium (80 *μ*L) and mixing thoroughly with trypan blue dye (10 *μ*L). An aliquot (10 *μ*L) of the mixture was then placed on a blood cell counting plate, and the total number of cells in the four large squares was counted. The cell concentration (cell number/mL cell suspension) was calculated as the total number of cells in four large squares/4 × 10^4^ × 10 (dilution multiple). Primary cell viability was assessed by comparing the numbers of dead cells (stained blue) and live cells (unstained). Two hundred primary liver cancer cells were counted, and the percentage of viable cells was calculated as the number of viable cells/total number of cells ×100%. Cell plating was carried out by counting cells in the logarithmic growth phase and adding them to a cell sample tank at a density of 1 × 10^5^ cells/mL. The cells were cultured in 384 well cell culture plates, and three compound wells were used for each drug. The volume in each well was 50 *μ*L and the number of cells was 5 × 10^3^. The cells were then cultured for 6–12 h.

#### 2.3.2. *In Vitro* HDS

**Figure 1:**
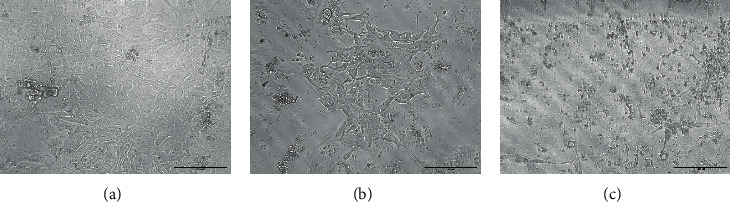
Primary liver cancer cells of control and test groups under the microscope. (a) Control group. (b) Inhibition rate: about 50%. (c) Inhibition rate: about 90%.

The primary cancer cells were divided into test and control groups and inoculated onto the drug screening plate. The test cells were treated with the planned drugs and combined drug regimens, and the control cells were treated with dimethyl sulfoxide (DMSO). All drugs were dissolved and diluted with DMSO. Cells were dosed at 0.1 *μ*L per well using an automatic work platform (JANUS® automated workstation; Perkin Elmer Inc., Wellesley, MA, USA) and incubated at 37°C, 5% CO_2_, for 72 h. CellTiter-Glo cell proliferation fluorescence detection reagent (10 *μ*L) was then added for 10 min, and the results were read using an Envision Plate Reader (Perkin Elmer Inc.). The inhibition rate was calculated as follows: inhibition rate = 100%-(RLU_Drug_ − RLU_Background_)/(RLU_DMSO_ − RLU_Background_) × 100%.  Figure 1 shows the primary liver cancer cells of the control and test groups under the microscope.

### 2.4. Test Drugs and Drug Susceptibility Analysis

The drugs used in this study included drugs recommended for the treatment of liver cancer by the National Comprehensive Cancer Network guidelines, and the drug dosages met the standards for clinical use. Other antitumor drugs were added to identify accurate and effective medications for the tested patients (Table 1). We used the 100% peak plasma concentration as the screening concentration to evaluate the inhibitory effect of the drug on cancer cells, which represented the drug's effect at the clinical dose. A higher inhibition rate indicated higher sensitivity of the patient's tumor cells to the drug. We defined an inhibition rate ≥80% as highly sensitive, <80% but ≥50% as moderate sensitivity, <50% but ≥25% as low sensitivity, and <25% as insensitive. We then classified cells as relatively sensitive or insensitive based on an inhibition rate ≥50%.

### 2.5. Immunohistochemistry of Tumor Specimens

All liver and liver tumor specimens were received by the Clinicopathology Department of the Molecular Medicine Testing Center of Chongqing Medical University for pathological diagnosis and immunohistochemical analysis. In this study, we examined the expression of alpha-fetoprotein (AFP), cytokeratin 7 (CK7), glypican 3 (GPC-3), antihepatocyte-specific antigen (HepPar-1), and Ki-67. The cells were divided into positive and negative groups for all antigens, except for Ki-67, and Ki-67 ≥ 20% was considered as high expression and <20% as low expression.

### 2.6. Statistical Analysis

Statistical analysis was performed using SPSS 24.0 (IBM Corp., Armonk, NY, USA). Measured data conforming to a normal distribution are presented as mean ± standard deviation, and measured data that did not conform to a normal distribution are presented as median (interquartile range). *In vitro* HDS results were presented as bar graphs. The relatively sensitive, relatively insensitive, positive (or high-Ki-67 expression), and negative (or low-Ki-67 expression) groups were analyzed by *χ*^2^ independence tests, and combinations of drugs and immunohistochemical markers with *P* < 0.05 in *χ*^2^ tests were further analyzed by Wilcoxon's rank-sum tests.

## 3. Results

### 3.1. Patient Information

The basic information on the enrolled patients is shown in Table 2.

### 3.2. Descriptive Statistics

**Figure 2:**
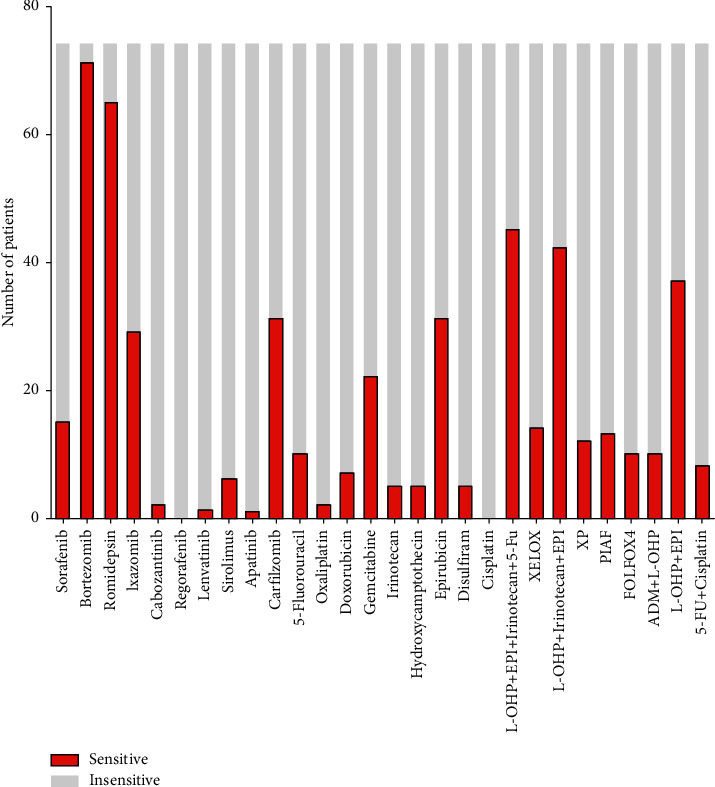
*In vitro* high-throughput drug sensitivity screening results.

**Figure 3:**
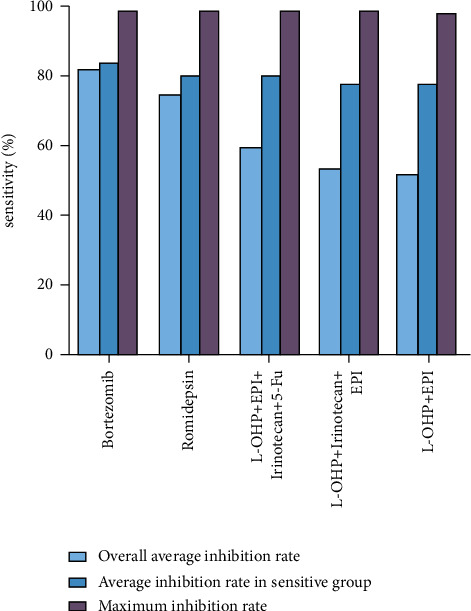
Inhibition rates of the five most effective drugs.

The sensitivities of liver cancer cells to the above 28 single drugs or combination regimens *in vitro* are shown in Figure 2. The sensitivity to most (19/28) single drugs or combination regimens was <50%, and no cells were sensitive to regorafenib or cisplatin. However, five drugs/combination regimens had sensitivity rates ≥50% (Figure 3 and Table 3).

### 3.3. Single Drugs

#### 3.3.1. Cytotoxic Drugs

The relationships between the sensitivities of liver cancer cells to the cytotoxic drugs and immunohistochemistry results are shown in Table 4. Because the inhibition rate for cisplatin was generally <50%, this agent was not tested. The results showed a significant correlation between AFP expression and gemcitabine sensitivity, with AFP-positive liver cancer cells being significantly more sensitive to gemcitabine than AFP-negative cells. In addition, CK7-positive liver cancer cells were significantly more susceptible to doxorubicin than CK7-negative cells. However, there were no significant correlations between sensitivity to any other cytotoxic drugs and immunohistochemistry results. Additional analysis with Wilcoxon's test confirmed that AFP-positive liver cancer cells (average rank: 38.50) were significantly more sensitive to gemcitabine than AFP-negative cells (average rank: 25.79) (*W* = 490.000, *Z* = −2.567, *P*=0.010). However, there was no significant difference in doxorubicin sensitivity between CK7-positive (average rank 36.29) and CK7-negative (average rank 30.16) liver cancer cells (*W* = 754.000, *Z* = −1.603, *P*=0.109).

#### 3.3.2. Targeted and Other Drugs

The relationships between the sensitivities of liver cancer cells to 10 targeted drugs and one other drug (disulfiram) are shown in Table 5. GPC-3 negative liver cancer cells were significantly more sensitive to cabozantinib than GPC-3-positive cells. However, there were no significant correlations between any other combinations and the immunohistochemistry results. Furthermore, Wilcoxon's test found no significant difference in sensitivity to cabozantinib between GPC-3 positive (average rank 32.27) and GPC-3-negative (average rank 35.63) liver cancer cells (*W* = 419.500, *Z* = −0.656, *P*=0.512).

### 3.4. Combination Regimens

The average inhibition rate of the combination regimens was mostly >50%, and we, therefore, performed *χ*^2^ tests on the nine combination regimens under the condition of a threshold inhibition rate of 80% (Table 6). AFP-positive liver cancer cells were significantly more sensitive to oxaliplatin (L-OHP) + epirubicin (EPI) + irinotecan + 5-fluorouracil (5-FU) and L-OHP + irinotecan + EPI. AFP-positive liver cancer cells were also more sensitive to the PIAF and L-OHP + EPI regimens than AFP-negative cells. There were no apparent correlations between the other regimens and immunohistochemistry markers. Wilcoxon's test confirmed that AFP-positive cells (average rank 38.39) were significantly more sensitive to L-OHP + EPI + irinotecan + 5-FU than AFP-negative cells (average rank 26.08) (*W* = 495.500, *Z* = −0.386, *P*=0.017) and were also significantly more sensitive to L-OHP + irinotecan + EPI and L-OHP + EPI (AFP-positive average ranks 38.73 and 38.08, respectively; AFP-negative average ranks 25.18 and 26.89, respectively). However, there was no significant difference in sensitivity to PIAF between AFP-positive and AFP-negative liver cancer cells according to Wilcoxon's test (*W* = 581.500, *Z* = −0.224, *P*=0.221).

**Figure 4:**
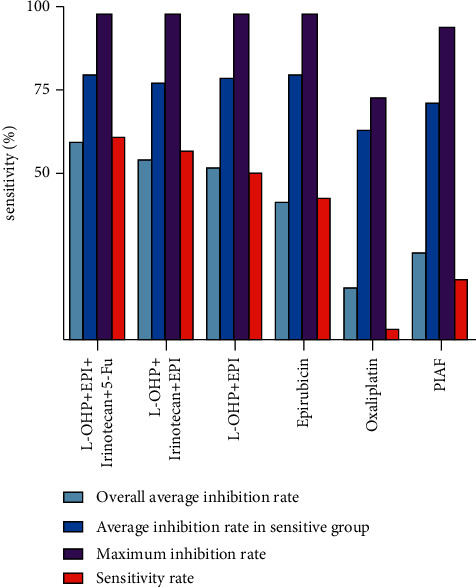
Comparison between medication regimens containing oxaliplatin and epirubicin with single agents.

We compared the inhibitory effects of the four regimens correlated with AFP according to *χ*^2^ tests and found that three regimens that contained L-OHP and EPI had similar impacts. All three were slightly better than EPI alone and significantly better than the PIAF regimen or L-OHP alone (Figure 4).

### 3.5. Relationships between Serum AFP, Liver Tumor AFP, and Drug Sensitivity

#### 3.5.1. Relationship between Serum AFP and Liver Tumor AFP

There was a significant correlation between serum AFP and liver tumor AFP (*χ*^2^ = 26.218, *P* < 0.001).

#### 3.5.2. Relationship between Serum AFP and Drug Sensitivity

The relationships between the four drugs or medication regimens and tumor AFP are shown in Table 7. According to *χ*^2^ tests, except for gemcitabine, there was a significant correlation between AFP and the three combination regimens (L-OHP + EPI + irinotecan + 5-FU, L-OHP + irinotecan + EPI, and L-OHP + EPI), with AFP-positive liver cancer cells being more sensitive than AFP-negative cells. Wilcoxon's test also showed that AFP-positive (average rank 45.69, 45.29, and 44.27) and AFP-negative (average rank 31.30, 31.53, and 32.11) liver cancer cells had significantly different sensitivities to the three combination regimens (*W* = 1440.000, *Z* = −2.802, *P*=0.005; *W* = 1450.500, *Z* = −2.679, *P*=0.007 and *W* = 1477.000, *Z* = −2.368, *P*=0.018), with AFP-positive liver cancer cells being more sensitive than AFP-negative cells.

## 4. Discussion

Liver transplantation and liver resection are the most effective treatments for liver cancer, but the lack of liver donors and the relatively low proportion of patients with surgical options have highlighted the need for nonradical surgical and systemic treatments and conversion therapy. Although interventional therapy, targeted therapy, and immunotherapy have all been applied in clinical practice, the occurrence of drug resistance and the high heterogeneity of liver cancer mean that the survival benefits of these treatments are not ideal. The diversification of cytotoxic, targeted, and immune drugs has increased the available treatment options for patients with advanced liver cancer; however, research into the personalized and targeted treatment of these patients remains in the preliminary stages. The current study aimed to identify immunohistochemical indicators that could guide clinical drug use and provide recommendations for future clinical trials.

We found that sole administration of the cytotoxic drugs commonly used in clinical practice had poor inhibitory effects on liver cancer cells *in vitro*, while some combination treatments had significantly better inhibitory effects. These results were in line with previous evaluations of cytotoxic drugs (e.g., doxorubicin [13], cisplatin [14], docetaxel [15], irinotecan [16], and 5-FU [17]) for the treatment of advanced HCC. These studies showed objective response rates of only 0%–10% for single cytotoxic drugs, with no survival benefit. Combination medications can significantly improve the inhibitory effect, and various tumors are clinically treated with multidrug chemotherapy regimens. In addition to sorafenib, we found the *in vitro* inhibitory effects on liver cancer cells of other targeted drugs currently approved by the FDA for the clinical treatment of HCC, such as lenvatinib, regorafenib cabozantinib, and apatinib, were not ideal. Combined with the mini-patient-derived xenograft results [18], similar to the current study, we considered the following possible explanations. The tumor cells in the *in vitro* HDS experiments did not form tissue masses and did not develop the corresponding tumor stroma and new blood vessels, which would lead to the failure of antiangiogenesis-targeted drugs. In addition, the observation period of the *in vitro* HDS experiments was short (about 1 week), and the onset of targeted drugs is slower. Although several single targeted drugs, such as bortezomib, romidepsin, and carfilzomib, have demonstrated outstanding *in vitro* inhibitory effects, there are no applications or experience in treating liver cancer to use as a reference, and the suitable dosage is therefore unclear. Moreover, *in vitro* HDS does not fully represent the *in vivo* effects, and our experiments could only indicate the potential values of the drugs and combinations, and further studies are needed to determine their safety and effectiveness.

Sorafenib is a tyrosine kinase inhibitor (TKI) that has been approved for the treatment of advanced HCC. It inhibits the Ras/Raf/MEK/ERK signaling pathway to inhibit tumor cell proliferation, platelet-derived growth factor receptor-*β*, vascular endothelial growth factor (VEGF) receptor-2, and hepatocyte factor receptor (c-Kit), thereby inhibiting tumor angiogenesis [19]. However, despite encouraging progress, sorafenib resistance remains a serious problem affecting treatment efficacy, with only about 30% of patients responding to sorafenib and most developing disease progression within 6 months [20]. We found that sorafenib demonstrated some effectiveness in *in vitro* tests (20.3%) compared with other targeted drugs used to treat liver cancer. We speculated that this was mainly due to its antitumor-proliferation effect. We then considered if it was possible to study the mechanism of sorafenib resistance using the *in vitro* susceptibility test. Numerous studies have examined the resistance mechanism of sorafenib. One study found that CD24 was a functional marker required for Akt/mTOR-mediated autophagy related to resistance to sorafenib. CD24 was highly expressed in sorafenib-resistant liver cancer cells, suggesting that it might be a potential target for HCC treatment [21]. Cancer metastasis, invasion, and growth are also affected by the tumor microenvironment, which is composed of various nonmalignant stromal cells. Recent studies [19] revealed complex multidirectional interactions between immune or nonimmune stromal cells and tumor cells during the development and progression of HCC. For example, hypoxia-inducible factor-2*α*, PT-2385, and PRMT6 enhanced the antitumor activity of sorafenib in liver tumors, while miR-338-3p promoted drug resistance via its interaction with sorafenib. Several studies have also explored serum cytokines as potential biomarkers for predicting sorafenib response, such as VEGF-A [22], angiopoietin-2 [23], and insulin-like growth factor-1 [24]. However, although the results indicated the potential prognostic values of these factors in HCC, their roles in predicting sorafenib response remain to be verified. Genetic alterations, such as single nucleotide polymorphisms (SNPs) in genes encoding proteins involved in the angiogenic process, have been studied as potential biomarkers for antiangiogenic therapy, and specific SNPs in the VEGF and VEGF receptor genes were associated with PFS and OS in HCC patients treated with sorafenib [25]. In addition to the Ras/Raf/MEK/ERK signaling pathway, other pathways, such as the mitogen-activated protein kinase (MAPK) pathway, also play an important role in the occurrence and development of HCC. Previous studies [26] showed that high-dose sorafenib and other TKIs inhibited the MAPK pathway, blocked tumor cell proliferation and viability, and induced cancer cell apoptosis, while low-dose TKIs increased MAPK signaling. This phenomenon could help to explain the unsatisfactory clinical results of sorafenib treatment. The BRAF pathway is also considered to play an important role in HCC; although it may not be the key to carcinogenesis, HCC patients with BRAF mutations may have more aggressive tumors and stronger resistance to TKI treatment. A long noncoding RNA involved in the BRAF pathway (BRAF lnc-RNA, i.e., BANCR) has been shown to play a key role in the acquired resistance of the MAPK pathway to TKI [27]. Multipathway inhibition may thus be a new direction for HCC treatments in the future [26]. Furthermore, the emerging research [28, 29] indicated that angiogenesis and immunosuppression are closely related and parallel mechanisms in tumor progression: the highly abnormal and impaired tumor vasculature actively promotes immunosuppression [30]. And proteins that play a major role in angiogenesis can also directly or indirectly affect the components of the immune system, ultimately resulting in immunosuppression [29]. The combination of antiangiogenesis therapy and immunotherapy seems to be possible to break the balance of the tumor microenvironment and improve the response to treatment. It could be a new direction for our future studies.

We found significant correlations between three single drug and four combination drug regimens and immunohistochemistry markers. Gemcitabine is a pyrimidine antimetabolite with extensive antitumor activity. It has demonstrated therapeutic effects against advanced HCC, with single-agent treatment being well-tolerated with a partial response rate of 17.8% in patients with advanced HCC [18, 31], as well as showing good safety [32, 33]. Gemcitabine showed a 29.7% inhibition rate in the current *in vitro* HDS and was significantly correlated with AFP expression; however, the specific mechanism responsible for gemcitabine's unique effect on AFP-positive liver cancer cells remains unknown. More studies are therefore warranted to investigate the mechanism of gemcitabine in the treatment of liver cancer. We also anticipate further studies to examine the therapeutic effects of gemcitabine, alone or in combination, on AFP-positive HCC patients, with the aim of improving the outcomes of liver cancer treatment.

CK7 is often considered an immunological marker for identifying intrahepatic cholangiocarcinoma and HCC. Previous studies [34] demonstrated positive expression of CK7 in HCC. CK7 may be derived from the malignant degeneration of hepatic progenitor cells, but its prognostic value in HCC is still unclear. Although the current study found a significant correlation between CK7 expression and doxorubicin sensitivity, there was no significant difference according to Wilcoxon's test. This may be because of the low inhibition rate of doxorubicin (9.46%, 7/74) and its poor inhibitory effect on liver cancer cells in *in vitro* HDS. Based on these results, we do not recommend further exploration of this relationship.

The results for cabozantinib were affected by the same problem; its 2.90% sensitivity rate (2/69) in the *in vitro* HDS was all due to GPC-3 negative cases, and Wilcoxon's test found no significant difference in inhibition rates between GPC-3-negative and GPC-3-positive liver cancer cells, suggesting that this relationship is unlikely to be significant. Cabozantinib is a multireceptor tyrosine kinase (RTK) inhibitor that can bind to and inhibit a variety of RTKs, including MET, RET, vascular endothelial growth factor-1, 2, and 3. Research [35] has proved that cabozantinib had an antiangiogenic effect, which could not be detected in the *in vitro* susceptibility test and which may thus explain the poor results for cabozantinib in the current test.

The sensitivities of liver cancer cells to combination regimens in the current study were all closely related to AFP. Of the four combination regimens correlated with AFP according to *χ*^2^ tests, three contained L-OHP and EPI. Although the PIAF regimen was the only combination regimen to pass the *χ*^2^ test that did not include these two drugs, Wilcoxon's test found no significant difference between the AFP-negative and AFP-positive groups in terms of sensitivity to this regimen. We compared the inhibitory effects of the four regimens with L-OHP and EPI. According to the results, we hypothesized that L-OHP and EPI exerted synergistic inhibitory effects in AFP-positive liver cancer cells. The AFP-positivity rate among all 79 HCC patients was only 27.54% (19/69), indicating that its reliability for the pathological diagnosis of HCC was not ideal. AFP is a glycoprotein derived from embryonic endoderm cells. It promotes the proliferation of liver cancer cells and the formation of tumor blood vessels and enhances the antiapoptotic effect of liver cancer cells [36]. EPI inhibits the activity of topoisomerase II*α* by inhibiting the cleavage of supercoiled DNA and inhibiting DNA transcription and replication and has been used to treat advanced HCC [37]. Compared with other anthracycline drugs (such as doxorubicin), EPI may cause fewer side effects (such as abnormal liver function and cardiotoxicity) [38, 39]. Previous reports of EPI combined with other platinum agents (such as cisplatin) for the treatment of liver cancer [40, 41] showed that it was relatively safe and effective for the treatment of advanced HCC. Furthermore, although the current study found that L-OHP was not effective *in vitro*, it has previously demonstrated outstanding results for the treatment of HCC. For example, the FOLFOX4 regimen, containing L-OHP and 5-FU, has passed the certification of a large-scale phase III clinical study [42], and the GEMOX regimen containing L-OHP and gemcitabine achieved an objective response rate of 22% and overall survival of 11 months [43]. The combination of L-OHP and EPI is often used in research into gastric cancer chemotherapy [44, 45]. However, its mechanism of action is still not clear, and it is rarely used for the treatment of liver cancer. The reported effectiveness and safety of the combination of L-OHP and EPI warrant further studies of their effects in the treatment of AFP-positive liver cancer.

Although the results of a few cases differed, liver tumor AFP and serum AFP showed a very high correlation. However, serum AFP showed a higher correlation with sensitivity to the three combination regimens compared with liver tumor AFP, suggesting that serum AFP may predict sensitivity to these combinations more accurately. Unfortunately, we have not yet retrieved the research on the mechanism of serum AFP affecting drug sensitivity. Further studies are planned to clarify this issue, and confirmation of the effect of serum AFP would be an important step toward the provision of personalized medical treatment.

The current study had the advantage of being harmless to the patients. The combined analysis of immunohistochemistry and *in vitro* HDS results in the enrolled patients has identified several potentially relevant drug-immunohistochemistry combinations, which may help to guide personalized treatments for patients with advanced liver cancer. Our study also had some limitations: this was a retrospective study, and the preliminary results and hypotheses thus need to be confirmed in clinical studies. In addition, the relatively small number of included cases may tune down the generability of our findings. More correlations may be detected in larger sample sizes, which could make the data more convincing. The success rate for the culture and expansion of primary tumor cells was relatively poor (about 30%–40%), and it was closely related to the size of the specimen. Patients with liver cancer without indications for surgery were therefore not suitable for inclusion in the study, even if liver cancer specimens could be obtained by a percutaneous liver puncture. The results of *in vitro* HDS cannot fully represent the drug's effect *in vivo*, and the absence of associated tissues and organs may make some drugs ineffective and exhibit low inhibition rates *in vitro*. And additional safety and efficacy tests are required before they can be entered into clinical trials. The current list of tested drugs could also be extended. More immunological drugs combined with cytotoxic drugs are available to treat HCC, such as sorafenib plus doxorubicin [46] and sorafenib combined with gemcitabine [34], indicating further potential for exploration in this field. Further studies are also needed to explore the synergistic effects of the drugs and their relationships with the corresponding immunohistochemistry markers.

## 5. Conclusion

The current results showed that gemcitabine had a greater inhibitory effect against AFP-positive compared with AFP-negative liver cancer cells *in vitro* HDS. Three combination regimens containing L-OHP and EPI (L-OHP + EPI + irinotecan + 5-FU, L-OHP + EPI, and L-OHP + irinotecan + EPI) also had greater inhibitory effects in AFP-positive than in AFP-negative liver cancer cells. And compared with liver tumor AFP, serum AFP may predict sensitivity to these combinations more accurately. These results may provide guidance for the future selection of personalized treatment strategies in patients with advanced liver cancer. However, further clinical studies are needed to confirm these results.

## Figures and Tables

**Table 1:** Test drug composition and dosage.

General Chemotherapeutic Drugs	5-Fluorouracil	300 mg/㎡	Combined chemotherapy regimens	L-OHP + EPI + Irinotecan+5-Fu	Oxaliplatin 130 mg/㎡ + epirubicin 90 mg/㎡ + irinotecan 350 mg/㎡ + 5-fluorouracil 400 mg/㎡
	Oxaliplatin	130 mg/㎡			
	Doxorubicin	75 mg/㎡			
	Gemcitabine	1,000 mg/㎡		XELOX	Capecitabine 1,250 mg/㎡ + oxaliplatin 130 mg/㎡
	Irinotecan	350 mg/㎡			
	Hydroxycamptothecin	6 mg		L-OHP + Irinotecan + EPI	Oxaliplatin 130 mg/㎡ + Irinotecan 350 mg/㎡ + epirubicin 90 mg/㎡
	Epirubicin	60 mg/㎡			
	Cisplatin	20 mg/㎡		XP	Capecitabine 2,000 mg/㎡ + cisplatin 60 mg/㎡

Molecular targeted drugs and others	Sorafenib	400 mg		PIAF	Cisplatin 20 mg/㎡ + doxorubicin 40 mg/㎡ + 5-fluorouracil 400 mg/㎡
	Bortezomib	1.3 mg/㎡			
	Romidepsin	10 mg/㎡			
	Ixazomib	4 mg		FOLFOX4	Oxaliplatin 85 mg/㎡ + 5-fluorouracil 600 mg/㎡
	Cabozantinib	60 mg			
	Regorafenib	160 mg		ADM + L-OHP	Doxorubicin 60 mg/㎡ + oxaliplatin 130 mg/㎡
	Lenvatinib	24 mg			
	Sirolimus	2 mg		L-OHP+EPI	Oxaliplatin 130 mg/㎡ + epirubicin 90 mg/㎡
	Apatinib	850 mg			
	Carfilzomib	20 mg/㎡		5-FU + Cisplatin	5-fluorouracil 160 mg/㎡ + cisplatin 6 mg/㎡
	Disulfiram	500 mg			

**Table 2:** Basic information on the enrolled patients (cases (%)).

Age (>55)	35 (47.3)	AFP (>400 ng/liter)	26 (35.1)
Gender (male)	65 (87.8)	Number of lesions (single)	66 (89.2)
Hepatitis B history	59 (79.7)	Maximum diameter of lesions (>5 cm)	33 (44.6)
Liver cirrhosis	32 (43.2)	Differentiation (poorly differentiated, undifferentiated)	19 (25.7)
Preoperative liver function (A)	72 (97.3)	BCLC staging (BCLC A)	36 (48.6)

**Table 3:** Inhibition rates of the five most effective drugs.

	Overall average inhibition rate	Average inhibition rate in sensitive group	Maximum inhibition rate
Bortezomib	82.00 ± 14.03	83.58 ± 11.93	98.66
Romidepsin	74.69 ± 17.48	79.49 ± 12.14	98.05
L-OHP + EPI + Irinotecan + 5-Fu	59.49 ± 27.37	79.48 ± 10.94	96.91
L-OHP + Irinotecan + EPI	53.72 ± 29.22	76.96 ± 12.19	97.71
L-OHP + EPI	51.55 ± 29.66	78.13 ± 12.61	96.92

**Table 4:** Relationships between immunohistochemistry markers and cytotoxic drug sensitivity.

	AFP		CK7		GPC-3		HepPar-1		Ki-67	
	*χ* ^2^	*P*	*χ* ^2^	*P*	*χ* ^2^	*P*	*χ* ^2^	*P*	*χ* ^2^	*P*
5-Fluorouracil	0.327	0.568	2.517	0.113	0.032	0.858	0.104	0.747	0	1.000
Oxaliplatin		0.478		1.000		1.000		1.000		0.505
Doxorubicin	0.261	0.609	4.002	0.045	0	1.000	0.093	0.761	0.240	0.625
Gemcitabine	6.102	0.014	1.146	0.284	0.182	0.670	0.621	0.430	0.459	0.498
Irinotecan	1.363	0.243	0	0.994		0.575	0	1.000	0	1.000
Hydroxycamptothecin	1.363	0.243	1.154	0.283		0.575	0	1.000	0.585	0.444
Epirubicin	1.210	0.271	0.053	0.819	0.206	0.650	0.219	0.640	0.009	0.925
Cisplatin	—	—	—	—	—	—	—	—	—	—

**Table 5:** Relationships between immunohistochemistry markers and sensitivity to targeted and other drugs.

	AFP		CK7		GPC-3		HepPar-1		Ki-67	
	*χ* ^2^	*P*	*χ* ^2^	*P*	*χ* ^2^	*P*	*χ* ^2^	*P*	*χ* ^2^	*P*
Sorafenib	0.187	0.666	1.218	0.270	0.759	0.384	1.499	0.221	0.165	0.684
Bortezomib		1.000	0.572	0.449		1.000		0.525		0.550
Romidepsin	0	1.000	1.895	0.169	0.000	0.994	0.002	0.969	0.013	0.908
Ixazomib	3.260	0.071	1.947	0.163	0.206	0.650	0.138	0.711	1.315	0.252
Cabozantinib		1.000		0.525		0.033		0.525		0.505
Regorafenib	—	—	—	—	—	—	—	—	—	—
Lenvatinib		1.000		1.000		0.188		1.000		0.294
Sirolimus	0.658	0.417	0	1.000	0	1.000	0.106	0.745	0	1.000
Apatinib		1.000		1.000		0.188		1.000		0.294
Carfilzomib	0.307	0.580	0.550	0.458	1.169	0.280	0.080	0.777	0.009	0.925
Disulfiram	0.016	0.898	2.468	0.116		0.575	0	1.000	1.102	0.294

**Table 6:** Relationships between immunohistochemistry markers and sensitivity to combination regimens.

	AFP		CK7		GPC-3		HepPar-1		Ki-67	
	*χ* ^2^	*P*	*χ* ^2^	*P*	*χ* ^2^	*P*	*χ* ^2^	*P*	*χ* ^2^	*P*
L-OHP + EPI + irinotecan + 5-fu	8.168	0.004	1.146	0.284	0.296	0.586	0.621	0.430	0.700	0.403
XELOX	2.601	0.107	0.216	0.642		1.000	0.092	0.761	0.585	0.444
L-OHP + irinotecan + EPI	5.705	0.017	3.222	0.073	0.003	0.956	0.299	0.584	0.609	0.435
XP	2.601	0.107	0.216	0.642		1.000	0.092	0.761	0.585	0.444
PIAF	4.871	0.027	1.154	0.283		0.575	0	1.000	0.980	0.322
FOLFOX4	2.601	0.107	0.216	0.642		1.000	0.092	0.761	0.585	0.444
ADM + L-OHP	0.211	0.646	0	0.994		1.000	0	1.000	0.585	0.444
L- + OHP + EPI	8.275	0.004	0.535	0.464	0.093	0.760	0.006	0.939	1.209	0.272
5-FU + Cisplatin		0.275	—	—		1.000		0.308		1.000

**Table 7:** Relationships between serum AFP and drug sensitivity.

	Serum AFP	
	*χ* ^2^	*P*
Gemcitabine	3.402	0.065
L-OHP + EPI + irinotecan + 5-Fu	7.251	0.007
L-OHP + irinotecan + EPI	9.712	0.002
L-OHP + EPI	10.017	0.002

## Data Availability

The HDS data used to support the findings of this study were supplied by the First Affiliated Hospital of Chongqing Medical University under license and so cannot be made freely available. Requests for access to these data should be made to shizr@hospital.cqmu.edu.cn (Dr. Shi).
